# Characterization of serine hydroxymethyltransferase GlyA as a potential source of D-alanine in *Chlamydia pneumoniae*

**DOI:** 10.3389/fcimb.2014.00019

**Published:** 2014-02-26

**Authors:** Stefania De Benedetti, Henrike Bühl, Ahmed Gaballah, Anna Klöckner, Christian Otten, Tanja Schneider, Hans-Georg Sahl, Beate Henrichfreise

**Affiliations:** Pharmaceutical Microbiology Section, Institute for Medical Microbiology, Immunology and Parasitology, University of BonnBonn, Germany

**Keywords:** chlamydial anomaly, persistence, aberrant bodies, D-alanine, alanine racemase, GlyA, penicillin, D-cycloserine

## Abstract

For intracellular *Chlamydiaceae*, there is no need to withstand osmotic challenges, and a functional cell wall has not been detected in these pathogens so far. Nevertheless, penicillin inhibits cell division in *Chlamydiaceae* resulting in enlarged aberrant bodies, a phenomenon known as chlamydial anomaly. D-alanine is a unique and essential component in the biosynthesis of bacterial cell walls. In free-living bacteria like *Escherichia coli*, penicillin-binding proteins such as monofunctional transpeptidases PBP2 and PBP3, the putative targets of penicillin in *Chlamydiaceae*, cross-link adjacent peptidoglycan strands via meso-diaminopimelic acid and D-Ala-D-Ala moieties of pentapeptide side chains. In the absence of genes coding for alanine racemase Alr and DadX homologs, the source of D-Ala and thus the presence of substrates for PBP2 and PBP3 activity in *Chlamydiaceae* has puzzled researchers for years. Interestingly, *Chlamydiaceae* genomes encode GlyA, a serine hydroxymethyltransferase that has been shown to exhibit slow racemization of D- and L-alanine as a side reaction in *E. coli*. We show that GlyA from *Chlamydia pneumoniae* can serve as a source of D-Ala. GlyA partially reversed the D-Ala auxotrophic phenotype of an *E. coli* racemase double mutant. Moreover, purified chlamydial GlyA had racemase activity on L-Ala *in vitro* and was inhibited by D-cycloserine, identifying GlyA, besides D-Ala ligase MurC/Ddl, as an additional target of this competitive inhibitor in *Chlamydiaceae*. Proof of D-Ala biosynthesis in *Chlamydiaceae* helps to clarify the structure of cell wall precursor lipid II and the role of chlamydial penicillin-binding proteins in the development of non-dividing aberrant chlamydial bodies and persistence in the presence of penicillin.

## Introduction

**Figure 1:**
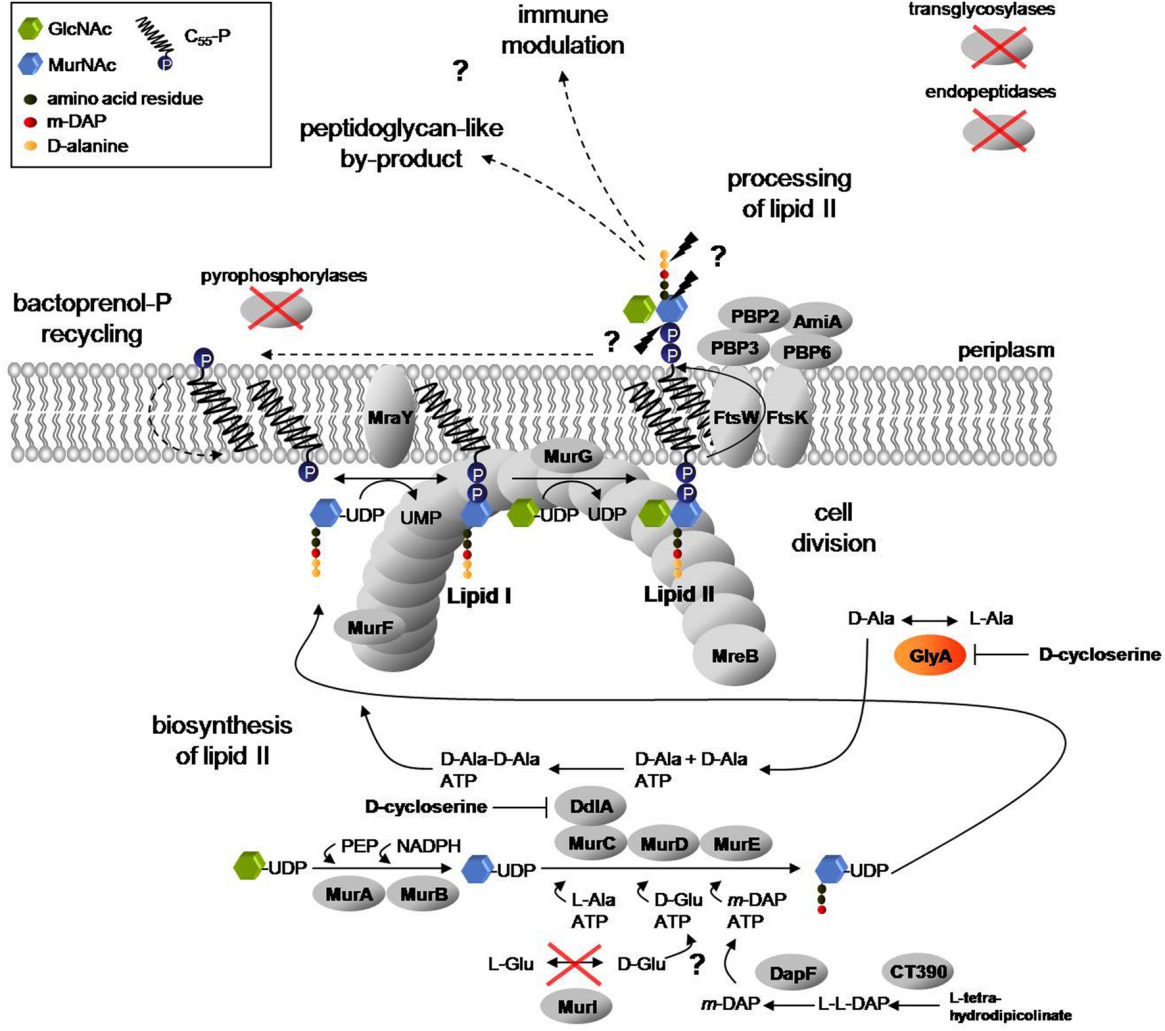
**Proposed lipid II pathway in *Chlamydiaceae***. A complete cycle of lipid II biosynthesis, including translocation to the periplasm, processing and bactoprenol carrier recycling is required for coordinated function of the divisome machinery and modulation of Nod1 and Nod2 mediated host immune response to chlamydial muropeptides. Biosynthesis of lipid II takes place in the cytoplasm and at the inner leaflet of the cytoplasmic membrane. In two consecutive biosynthesis steps of L-Ala racemization and D-Ala ligation, catalyzed by GlyA and the MurC/Ddl fusion protein, the D-Ala-D-Ala dipeptide is produced. MurF adds the dipeptide to the nascent peptide chain to complete synthesis of the soluble precursor UDP-MurNAc-pentapeptide and to provide D-Ala-D-Ala moieties in the cell wall precursors for transpeptidation activity of the penicillin-binding proteins PBP2 and PBP3. Actin-ortholog MreB functionally organizes MurF, MraY, and MurG (Gaballah et al., 2011), the last three enzymes in lipid II biosynthesis, at the septum. The synthesized precursor is translocated to the periplasm and processed by the concerted activity of the PBP enzymes and amidase AmiA to allow for bactoprenol-P recycling. In the process, the rudimentary by-product found by Liechti et al. (2013), in which the peptide side chains are cross-linked by peptide bonds, might result (Ghuysen and Goffin, 1999) and released muropeptides might contribute to modulation of the host immune response (McCoy and Maurelli, 2006). *Chlamydiaceae* lack transglycosylases as well as endopeptidases and pyrophosphorylases described so far to link lipid II sugar units to form glycan chains, to cleave peptide bridges between cross-linked glycan chains and to dephosphorylate bactoprenol-PP, respectively. Moreover, the L-Glu racemase MurI is absent. Question marks and dashed arrows highlight steps of the pathway that remain to be clarified. (GlcNAc: N-acetylglucosamine; MurNAc: N-acetylmuramic acid).

Acute and chronic diseases caused by *Chlamydiaceae* are a global health problem. The Gram-negative obligate intracellular pathogens depend on eukaryotic host cells to maintain their unique biphasic developmental cycle. One elusive phenomenon of the chlamydial biology has fascinated researches for two decades: for endobacteria, such as *Chlamydiaceae*, there is no need to resist osmotic challenges and a functional cell wall has not been detected in these pathogens so far (McCoy and Maurelli, 2006). Nevertheless, in the evolutionary process of adaptation to the host environment, *Chlamydiaceae* species conserved in their reduced genomes a nearly complete cell wall precursor biosynthesis pathway (Figure 1) and antibiotics that target cell wall biosynthesis are active (McCoy and Maurelli, 2006). Penicillin has no bactericidal effect, as seen in free-living bacteria, but induces a reversible state of persistence in *Chlamydiaceae* that is characterized by the formation of viable, enlarged, reticulate bodies. These persisting cells are called aberrant bodies (AB) and show resistance to azithromycin (Wyrick and Knight, 2004), the first-line treatment for chlamydial infections (CDC, 2010). Beta-lactam induced formation of non-dividing ABs has been observed in cell culture (Skilton et al., 2009) as well as *in vivo* (Phillips Campbell et al., 2012).

In free-living bacteria, cell division must be highly coordinated with cell wall biosynthesis to maintain cell integrity. The need for tightly interconnecting both cell biological processes may be reflected by the partial overlap of components from both multi-protein machineries; e.g., the transpeptidase PBP3 (FtsI) is essential for the incorporation of cell wall building blocks at the septal cell wall and for cell division.

The bacterial cell wall consists of peptidoglycan, a polymer of long chains with alternating sugar units of N-acetylglucosamine (GlcNAc) and N-acetylmuramic acid (MurNAc), which are cross-linked via flexible peptide bridges. Peptidoglycan is found in all eubacteria with the exception of some obligate intracellular species. Biosynthesis of peptidoglycan takes place in three stages (Figure 1). In the cytoplasm, six enzymes (MurA to MurF) catalyze the formation of the soluble precursor UDP-MurNAc-pentapeptide. Notably, the precursor contains D-Ala in positions 4 and 5 of the pentapeptide moiety. D-alanine is a unique and essential component in the biosynthesis of bacterial cell walls. The non-proteinogenic amino acid is synthesized by alanine racemases Alr and DadX and ligated by Ddl to form D-Ala-D-Ala. The dipeptide is attached to the amino acid in position 3 by the action of MurF to complete the pentapeptide side chain. In the first membrane-linked step, MraY catalyzes the synthesis of lipid I by transferring UDP-MurNAc-pentapeptide to the lipid carrier bactoprenol-phosphate (undecaprenyl-P). With the addition of UDP-GlcNAc, MurG synthesizes lipid II, the completed peptidoglycan cell wall building block. Lipid II is then translocated by the flippase FtsW to the outside of the cell and incorporated into the peptidoglycan network by the action of penicillin-binding proteins (PBPs) which exhibit transglycosylase and DD-transpeptidase activities.

The human cytosolic Pattern Recognition Receptors, Nod1 and Nod2, sensing bacterial cell wall fragments, recognize intracellular *C. pneumoniae* and subsequently mediate activation of the transcription factor NFkB which plays a key role in regulating the immune response to infection (McCoy and Maurelli, 2006). Nod1 and Nod2 receptor mediated recognition, together with the susceptibilty to penicillin, suggests that cell wall precursors/peptidoglycan fragments are synthesized by *Chlamydiaceae* during infection.

A nearly complete lipid II biosynthesis pathway has been found in genomes of *Chlamydiaceae* (Figure 1) (McCoy and Maurelli, 2006), and functional conservation of enzymes catalyzing cytoplasmic steps (MurA, MurC/Ddl, CT390, DapF, MurE, MurF) and the two membrane-linked steps (MraY and MurG) of cell wall precursor biosynthesis has been demonstrated (McCoy and Maurelli, 2006; McCoy et al., 2006; Henrichfreise et al., 2009; Patin et al., 2009, 2012). *Chlamydiaceae* genomes code for only two PBPs that serve as DD-transpeptidases in free-living bacteria. PBP2 and PBP3 are the putative targets of penicillin in *Chlamydiaceae* and cross-link adjacent peptidoglycan strands via meso-diaminopimelic acid and D-Ala-D-Ala moieties in *E. coli*. AmiA is the only ortholog of septal peptidoglycan hydrolyzing amidases found in chlamydial genomes. Moreover, *Chlamydiaceae* harbor a rudimentary set of cell division proteins, lacking the central organizer FtsZ, but comprising FtsW, FtsI (PBP3) and FtsK, and possess, despite their spherical shape, the cytoskeletal protein MreB (Gaballah et al., 2011). Chlamydial MreB was shown to interact with key components in lipid II biosynthesis and FtsK (Gaballah et al., 2011; Ouellette et al., 2012).

We proposed that retaining biosynthesis of lipid II in cell wall-lacking “minimal bacteria,” like *Chlamydiaceae*, may reflect a vital role of the lipid II pathway in prokaryotic cell division (Henrichfreise et al., 2009). Moreover, we discussed a model for the role of the conserved lipid II pathway in maintaining a functional divisome and contributing to modulation of host response in *Chlamydiaceae* (Figure 1). A recent study revealed the presence of cell wall sacculi in *Protochlamydia*, a genus of evolutionary older amoeba symbionts with less reduced genomes as compared to *Chlamydiaceae* (Pilhofer et al., 2013). In the pathogenic *Chlamydiaceae*, however, no functional cell wall but ring-like shaped structures were found and supposed to contain peptidoglycan-like material and localize to the division plane (Liechti et al., 2013). These findings are consistent with our model described above which implicates a crucial function of the PBP2 and PBP3 DD-transpeptidase activity in lipid II processing and sustaining a complete cycle of lipid II biosynthesis and recycling. PBP catalyzed DD-transpeptidation depends on the presence of the D-Ala-D-Ala terminus in the pentapeptide side chain of cell wall building blocks. Genomes of *Chlamydiaceae* do not encode homologs of the pyridoxal-5′-phosphate (PLP) cofactor requiring alanine racemases Alr and DadX. Therefore, the source of D-Ala and thus the presence of substrates for PBP2 and PBP3 activity in *Chlamydiaceae* have remained unclear for years.

We searched chlamydial genomes for genes encoding other PLP dependent proteins and found serine hydroxymethyltransferase GlyA to be conserved in all chlamydial genera. Serine hydroxymethyltransferases are found in eu- and prokaryotes and are well known for their function in reversible interconversion of serine and glycine using tetrahydrofolate as the one-carbon carrier. In addition, the enzymes show a particularly broad reaction specificity and catalyze other side reactions typical for PLP dependent enzymes, such as decarboxylation, transamination and retroaldol cleavage (Contestabile et al., 2001). Moreover, an alanine racemase co-activity was proven *in vitro* for GlyA from *E. coli* (Shostak and Schirch, 1988).

The aim of this study was to analyze GlyA as a potential source of D-Ala in *Chlamydiaceae*.

Here, we demonstrate that GlyA from *C. pneumoniae* is capable of the racemization of alanine *in vivo* and *in vitro* implicating that the enzyme can substitute for the absent alanine racemases and that D-Ala is self-synthesized in *Chlamydiaceae.*

## Results

### Racemization of alanine in chlamydiae

Using BLAST alignments, we searched *Chlamydiaceae* and enviromental chlamydiae genomes to identify genes coding for orthologs of *E. coli* PLP cofactor-requiring enzymes known to confer alanine racemization activity (Table 1). In contrast to the *Chlamydiaceae* and *Simkania*, the three environmental chlamydiae genera *Parachlamydia, Protochlamydia*, and *Waddlia* harbored one ortholog of the Alr or DadX alanine racemases. GlyA was the only enzyme to be encoded in *Chlamydiaceae* and in all enviromental chlamydiae.

**Table 1:** **PLP cofactor-requiring enzymes involved in biosynthesis of D-Ala**.

***E. coli***	**Cpn**	**Ctr**	**Pac**	**Pam**	**Wch**	**Sne**
Alr (alanine racemase)	–	–	–	pc0631 (3e–34)	wcw_0679 (1e–32)	–
DadX (alanine racemase 2)	–	–	PUV_23750 (2e–28)	–	–	–
GlyA (serine hydroxymethyltransferase)	CPn0521 (2e–107)	CT432 (2e–108)	PUV_05830 (6e–118)	pc0444 (3e–107)	wcw_1457 (2e–117)	SNE_A20 270 (1e–114)
MetC (cystathionine beta-lyase)	–	–	PUV_18690 (2e–40)	–	wcw_1145 (4e–40)	–

Locus tags of genes coding for enzymes involved in biosynthesis of D-Ala are shown for two exemplary Chlamydiaceae and for environmental chlamydiae species. The expected (E) values of BLAST P alignments are listed in brackets. E. coli, Escherichia coli W3110 (NC_007779.1); Cpn, Chlamydia pneumoniae CWL029 (NC_000922); Ctr, Chlamydia trachomatis D/UW-3/Cx (NC_000117); Pac, Parachlamydia acanthamoebae UV-7 (NC_015702.1); Pam, Protochlamydia amoebophila UWE25 (NC_005861.1); Wch, Waddlia chondrophila WSU 86-1044 (NC_014225.1); Sne, Simkania negevensis Z (NC_015713.1).

### *In vivo* activity of GlyA from *C. pneumoniae*

**Figure 2:**
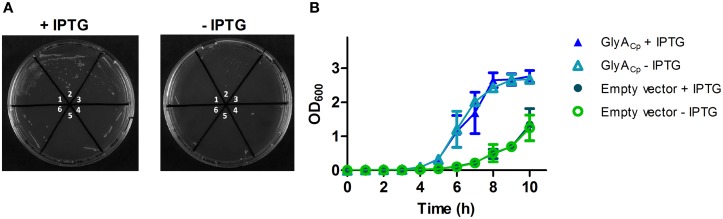
**GlyA_Cp_ exhibits *in vivo* activity in an *E. coli* racemase double mutant**. A temperature sensitive Δ *alr*Δ *dadX E. coli* double mutant was transformed with pET21b-*glyA*_Cp_ to allow for the expression of GlyA_Cp_ in the cytoplasm. Independently generated transformants (1–3 containing pET21b-*glyA*_Cp_ and 4–6 containing the empty vector) were grown on solid **(A)** or in liquid **(B)** LB medium under limited D-Ala growth conditions at 42°C. LB medium was supplemented with 5 mg/L D-Ala, 50 μM of cofactor PLP, 25 μg/ml thymine and 50 μg/ml ampicillin. Expression of GlyA_Cp_ was induced by the addition of 0.1 mM IPTG.

In the absence of a tractable system to genetically manipulate *C. pneumoniae* we tested whether heterologous expressed *C. pneumoniae* GlyA (GlyA_Cp_) shows an effect on the D-Ala auxotrophic phenotype of an *E. coli* Δ *alr*Δ*dadX* racemase double mutant strain. Our experiments in liquid and solid culture revealed that chlamydial GlyA did not completely reverse the need of exogenous D-Ala of the racemase mutant strain but favored its growth under D-Ala limited conditions (Figure 2). These findings suggest that GlyA_Cp_ is a functional alanine racemase and capable of generating D-Ala in *E. coli*.

### Recombinant GlyA_Cp_ has L-alanine racemase activity

**Figure 3:**
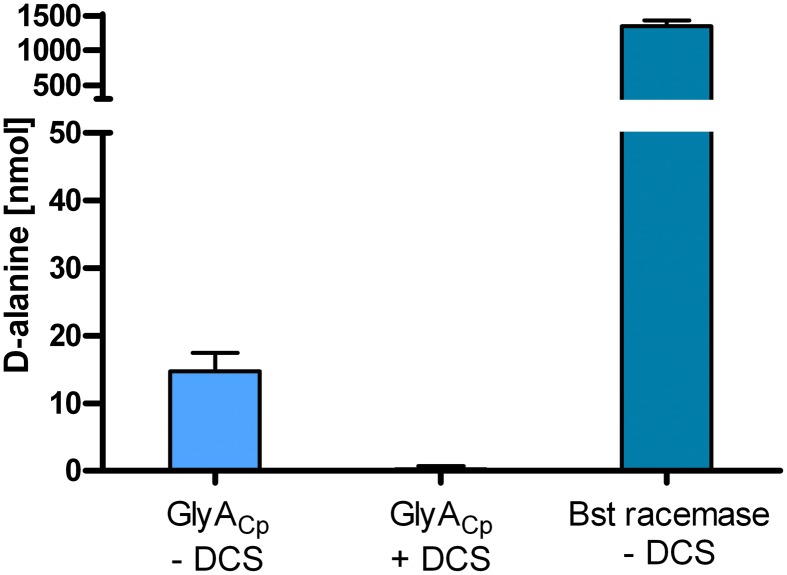
**The purified serine hydroxymethyltransferase GlyA_Cp_ has alanine racemase activity**. *In vitro* activity of recombinant GlyA_Cp_ was tested in a coupled enzymatic assay containing L-Ala and cofactor PLP. D-Ala that was produced by 1 μg GlyA or Bst racemase was converted to pyruvate by the activity of DAAO and colorimetrically quantified. (DCS: D-cycloserine; Bst racemase: alanine racemase from *B. stearothermophilus*).

To investigate the potential alanine racemase activity of the serine hydroxymethyltransferase GlyA from *C. pneumoniae*, we overexpressed recombinant GlyA_Cp_ in *E. coli* and purified the Strep-tagged protein. *In vitro* activity of GlyA_Cp_ was tested in a D-amino acid oxidase coupled enzymatic assay containing L-Ala and cofactor PLP. Alanine racemase from *Bacillus stearothermophilus* served as positive control. D-Ala that was produced by GlyA was converted to pyruvate by the activity of D-amino acid oxidase (DAAO) and colorimetrically quantified. The chlamydial GlyA converted L-Ala to D-Ala *in vitro* exhibiting weak racemase activity in comparision to the enzyme from *B. stearothermophilus* (Figure 3).

### GlyA_Cp_ racemase activity is sensitive to D-cycloserine

D-cycloserine is a structural analog of D-Ala and competitively inhibits activity of alanine racemases and D-Ala ligases from free-living bacteria (Strominger et al., 1960; Lambert and Neuhaus, 1972). The inhibitor has anti-chlamydial activity in chick embryo yolk sac infection (Moulder et al., 1963) which can be reversed by the addition of D-Ala. For the *C. trachomatis* D-Ala ligase Ddl which is encoded as a fusion with MurC, as typical for *Chlamydiaceae*, sensitivity to D-cycloserine has been proven before (McCoy and Maurelli, 2005). We performed activity assays for GlyA_Cp_ in the presence of D-cycloserine and identified the enzyme to be a second target of D-cycloserine in *Chlamydiaceae* (Figure 3).

## Discussion

The source of D-Ala in *Chlamydiaceae* and thus the presence of the transpeptidation substrates for PBPs is a crucial aspect of the chlamydial anomaly that will help to gain understanding of the penicillin induced persistence in these human pathogens. Penicillin and other beta-lactams structurally mimic the D-Ala-D-Ala terminus of the pentapeptide side chain of the lipid II cell wall building blocks and are recognized by the active sites of DD-transpeptidase PBPs. Beta-lactams are active against *Chlamydiaceae* in cell culture (McCoy and Maurelli, 2006; Skilton et al., 2009) and *in vivo* (Phillips Campbell et al., 2012) and the chlamydial monofunctional DD-transpeptidases PBP2 and PBP3, recovered in detergent-soluble fractions from whole cell preparations, bind [H^3^]-penicillin (Barbour et al., 1982). Moreover, the recombinant MurC/Ddl protein from *C. trachomatis* has been shown to specifically ligate D-Ala but not the L-Ala enantiomer to form alanine dipeptides and MurF was demonstrated to add these D-Ala-D-Ala dipeptides to the lipid II peptide side chain (McCoy and Maurelli, 2006; Patin et al., 2012). Moulder et al. (1963) proved anti-chlamydial activity of D-cycloserine in chick embryo yolk sac infection models and demonstrated the specific reversal of this effect by the addition of D-alanine (Moulder et al., 1963). D-cycloserine is a structural analog of D-Ala and well known as competitive inhibitor of alanine racemases and the D-Ala ligase Ddl in other bacteria (Strominger et al., 1960; Lambert and Neuhaus, 1972). Finally, feeding of replicating *Chlamydia trachomatis* with D-Ala-D-Ala probes revealed evidence for the incorporation of D-Ala into ring-like shaped peptidoglycan-like structures (Liechti et al., 2013). All in all, these data strongly indicate that D-Ala is present in the cells of *Chlamydiaceae* and plays an essential role in chlamydial cell biology. In the past, the mammalian host was discussed as a potential source of D-Ala (McCoy and Maurelli, 2006) and in *Chlamydiaceae* genomes a D-alanine permease (DagA_2) was annotated (Stephens et al., 1998) that could allow for the passive transport of D-Ala over the chlamydial cytoplasmic membrane. Though D-Ala is found almost exclusively in the microbial world and is not synthesized in mammalian cells, D-Ala was detected in trace quantities in mammalian urine and tissues, apparently due to breakdown products from intestinal and food bacteria (Guoyao, 2013). Nevertheless, experiments with *Listeria monocytogenes* and *Shigella flexneri* indicated that mammalian host cells cannot serve as source of D-Ala. Alanine racemase knockout mutants of both facultative intracellular species failed to survive within mammalian host cells unless exogenous D-Ala was added to the cell culture medium (McCoy and Maurelli, 2006). Based on these data, self-biosynthesis of D-Ala by alternative racemase activity is more likely the source of D-Ala in *Chlamydiaceae*.

For *E. coli*, besides the constitutive expressed Alr racemase and the catabolic DadX racemase, two other PLP dependent enzymes involved in the methionine pathway have been shown to confer L-Ala racemization as a side reaction. Overexpressed cystathionine beta-lyase MetC completely reversed the D-Ala auxotrophic phenotype of an *E. coli* racemase double mutant whereas racemase co-activity of serine hydroxymethyltransferase GlyA was not sufficient to allow for growth on D-Ala lacking medium (Kang et al., 2011). Recently, *in vitro* activity of MetC from *Wolbachia* indicated that the enzyme substitute for the absent alanine racemases in these endosymbionts of many arthropods and filarial nematodes. *Wolbachia* are excellent targets for anti-filarial therapy. Similar to *Chlamydiaceae*, these obligate intracellular bacteria lack a cell wall but treatment with lipid II biosynthesis blocking fosfomycin, results in enlarged *Wolbachia* cells (Vollmer et al., 2013). Among the *Chlamydiales* only the environmental *Parachlamydia* and *Waddlia* possess a MetC ortholog but all chlamydial genera harbor GlyA. The serine hydroxymethyltransferase is the only component of the methionine pathway that is encoded by *Chlamydiaceae* genomes and phylogenetic analysis indicated lateral transfer of the *glyA* gene from Actinobacteria to the common ancestor of *Chlamydiales* (Griffiths and Gupta, 2006). Moreover, transcription profiles revealed overlapping expression of the genes encoding GlyA, enzymes for lipid II biosynthesis (MurA to MurF, MraY, and MurG) as well as processing (PBP2, PBP3, and AmiA), the structural protein MreB, and the cell division proteins FtsW and FtsK (Belland et al., 2003). These data suggest an essential function of GlyA in chlamydial biology and correlation with the cellular processes of lipid II biosynthesis and cytokinesis.

We demonstrated L-Ala racemase activity for the GlyA enzyme from *C. pneumoniae in vivo* in an *E. coli* racemase double mutant and characterized the purified protein *in vitro*. Moreover, we identified GlyA as a second target of D-cycloserine besides MurC/Ddl in *Chlamydiaceae.*

Our results implicate that the enzyme can substitute for the absent alanine racemases and that D-Ala is present and self-synthesized in *Chlamydiaceae*. The observed weak alanine racemase activity of GlyA_Cp_ cannot completely compensate D-Ala requirements of the *E. coli* racemase mutant to build a functional cell wall but might be sufficient to produce D-Ala in amounts that maintain the lipid II biosynthesis pathway in the cell wall-lacking *Chlamydiaceae* for the proposed functions of the cell wall precursor in co-ordination of cell division and modulation of the host immune response. Future research toward the elucidation of the chlamydial anomaly will include the isolation and structural characterization of lipid II building blocks and biochemical analysis of the penicillin binding proteins PBP2 and PBP3, the putative targets of penicillin whose activity depends on the presence of D-Ala-D-Ala in the pentapeptide side chain of lipid II.

Like other effectors, such as interferon-γ and tumor necrosis factor-α, penicillin can be used to induce persistence as an experimental tool to study chlamydiae/host interactions. Knowledge of the underlying mechanisms of penicillin induced formation of ABs will help to assess these results on the pathogenicity of *Chlamydiaceae*.

Moreover, analysis of the molecular biology of penicillin induced persistence is important to improve understanding of long-term infection in patients in particular as to the role of chlamydial cell wall precursors in immune modulation and refractory of ABs to anti-chlamydial agents.

## Materials and methods

### Bacterial strains and growth conditions

*E. coli* JM83 harboring the GlyA_Cp_ expression vector was grown on Luria Bertani (LB) agar plates containing 30 μg/ml chloramphenicol and 100 μg/ml ampicillin, respectively. The temperature sensitive *E. coli* Δ *alr*Δ *dadX* racemase double mutant TKL-10 was maintained on LB agar plates containing 25 μg/ml thymine.

### *In vivo* complementation

*E. coli* TKL-10 Δ *alr*Δ *dadX* was transformed with pET21b-*glyA*_Cp_ and grown in liquid or on solid LB medium supplemented with 5 mg/L D-Ala, 50 μM PLP, 25 μg/ml thymine, 50 μg/ml ampicillin and 0.1 mM IPTG at 42°C. For each experiment three independently generated transformants were used and controls with *E. coli* TKL-10 Δ *alr*Δ *dadX* harboring the empty pET21b vector were carried out.

### Cloning of *glyA*

The *glyA* gene from *C. pneumoniae* GiD was amplified by PCR using primer glyACp_f (5′-ATGGTAGGTCTCAGGCCTTGCTAAAAGTTTTTGAGAAATTTAAGA-3′) and glyACp_r (5′-ATGGTAGGTCTCAGCGCTAACTAAAGCTTCTAAATCAATTTCAGG-3′) and cloned into pASK-IBA2c (IBA, Germany) using the BsaI restriction site to generate an N-terminal OmpA-leader peptide fused, C-terminal Strep-tagged protein for periplasmic overproduction. For cytoplasmic expression in complementation assays, *glyA* was amplified with primers glyACp_pET21_f (5′-CGTCTTTAGAAGCATATGCTAAAAG-3′) and glyACp_pET21_r (5′-GTCTCTGCGGCCGCAACTAAAGCTTC-3′) and cloned into pET21b (Novagen, VWR, Germany) using NdeI and NotI restriction sites.

### Overproduction and purification of GlyA_Cp_

*E. coli* JM83 cells, transformed with pASK-IBA2c-*glyA*_Cp_, were grown in no salt LB in presence of 30 μg/ml of chloramphenicol, 250 mM sucrose and 50 mM L-serine at 30°C. After induction at an OD_600_ of 1.2 with 200 ng/ml anhydrotetracyline (AHT), 50 μM PLP and 200 μM of folinic acid were added and the cells were incubated for 4 h at 25°C. The purification of GlyA_Cp_ was performed using the protocol for cleared lysates recommended by the manufacturer (IBA, Germany) with small modifications: the buffers contained 2% N-lauroylsarcosine (or 0.1% N-lauroylsarcosine in the washing and elution buffer), 2 mM 1,4-dithiothreitol (DTT) and 50 μM PLP. Purity of the protein was controlled using SDS-PAGE.

### *In vitro* GlyA_Cp_ activity assay

Racemase activity of GlyA_Cp_ was determined in a DAAO coupled enzymatic assay system as described previously with slight modifications (Francois and Kappock, 2007). Briefly, 1 μg GlyA_Cp_ or DAAO were incubated with 50 mM L-alanine in a final volume of 60 μl for 16 h at 37°C in 50 mM KH_2_PO_4_, pH 8, 100 mM KCl, 80 μM PLP and 2 mM DTT. D-Ala that was derived from GlyA_Cp_ racemization was deaminated into pyruvate by the activity of DAAO and indirectly quantified by determining the amount of produced pyruvate with a colorimetric assay using 2,4-dinitrophenylhydrazine (DNPH) as described before (Milner and Wood, 1976). Alanine racemase from *B. stearothermophilus* (Sigma-Aldrich, Germany) was used as positive control. PLP containing enzymes have been described to show weak transamination activity converting alanine to pyruvate. As a control for potential L-Ala transamination activity of GlyA_Cp_, we ran experiments in the absence of DAAO. No GlyA_Cp_ catalyzed production of pyruvate was detected. For GlyA_Cp_ inhibition assays, 10 mM D-cycloserine was added and both enzymatic steps were carried out consecutively with a step of heat deactivation in between as DAAO is sensitive to D-cycloserine inhibition.

### Conflict of interest statement

The authors declare that the research was conducted in the absence of any commercial or financial relationships that could be construed as a potential conflict of interest.
